# Value of Apoptotic, Antiapoptotic, and Cell Proliferation Markers in the Treatment of Graves' Disease

**DOI:** 10.1155/2018/3171280

**Published:** 2018-06-19

**Authors:** Jessica Castro de Vasconcelos, Icléia Siqueira Barreto, Patrícia Sabino Matos, Frederico Fernandes Ribeiro Maia, Marcos Antônio Tambascia, Maria Cândida Ribeiro Parisi, Denise Engelbrecht Zantut-Wittmann

**Affiliations:** ^1^Endocrinology Division, Department of Internal Medicine, University of Campinas, Campinas, SP, Brazil; ^2^Department of Pathology, Faculty of Medical Sciences, University of Campinas, Campinas, SP, Brazil

## Abstract

To better understand the genesis of autoimmunity in Graves' disease (GD), it is essential to study the mechanism of apoptosis and cell proliferation in thyroid cells and intrathyroidal lymphocytic infiltrate of GD patients. *Methods*. A cross sectional, observational study performed by evaluating histopathological samples of thyroidectomy products from GD patients using immunohistochemistry. New histological sections were prepared for immunohistochemical analysis with markers of cell proliferation, antiproliferation, apoptosis, and antiapoptosis. *Results*. Patients with GD who underwent radioiodine therapy (RIT) had a lower lymphocytic expression level of p27Kip1, and those who took beta-blockers had higher expression levels of BID (*BH3*-interacting domain) and a lower Ki-67 expression level in thyrocytes than those who did not. The association of a shorter diagnostic time with a lower expression level of MCL-1 in thyroid cells suggests that the hyperthyroid state was related to a lower antiapoptotic effect on thyrocytes. In comparison to patients with GD not using antithyroid drugs (ATD), we found a lower expression level of BID in lymphocytes for those who used ATD. *Conclusion*. In GD, the hyperthyroid state was associated with a lower antiapoptotic effect on thyroid cells. RIT, beta-blockers, and thionamide act by stimulating apoptosis of thyrocytes by intrathyroidal lymphocytes.

## 1. Introduction

Graves' disease (GD) is an autoimmune thyroid disease that involves [1, 2] autoreactivity against thyrocytes by antibodies, lytic granules released from cytotoxic T-lymphocytes (CTLs), and apoptosis and the inappropriate presence of death receptors that activate apoptotic pathways [3–5]. Death receptors and their ligands, as Fas or CD95 and Fas ligand (FasL), are inactive in a normal thyroid. However, under proinflammatory conditions, proapoptotic molecules are activated [6, 7]. Thyrocytes from GD patients express lower Fas levels creating an antiapoptotic environment. FasL-positive GD thyrocytes appear to induce Fas-mediated apoptosis of lymphocytes [8, 9].

BCL-2 (B-cell lymphoma/leukemia 2) is an antiapoptotic molecule from the BCL-2 protein family. Other molecules of the family participate in the control of apoptosis: antiapoptotic MCL-1 (myeloid cell leukemia 1) and proapoptotic BID (*BH3*-interacting domain). In patients with GD, cytokines promote humoural immunity predominance and upregulate BCL-2 in thyrocytes, which escape from apoptosis. However, this safety is not present in GD intrathyroidal lymphocytes [7, 10–12]. MCL-1 is relevant for the survival of cell lineages of lymphocytes, stem cells, and neutrophils [13, 14]. BID has lower expression levels in normal thyroid cells. However, when thyroid cells are exposed to inflammatory cytokines, there is BID overexpression. In mice with BID overexpression, the risk of developing autoimmune thyroiditis, induced by known factors such as iodine, was higher than in controls [10, 15, 16].

Autoimmune thyroid diseases and their clinical characteristics are distinguished by an imbalance between the degree of apoptosis and cell proliferation. Ki-67 and p27Kip1 proteins are markers related to cell proliferation in thyroid cells. Ki-67 is a nuclear protein produced in dividing cells that is used as a marker to identify active dividing cells. Doganay et al. [17] determined Ki-67 major expression in the follicles adjacent to lymphocytic infiltrate associated with regenerative hyperplasia; and some authors observed higher expression levels of Ki-67 in GD than nodular goiter [18, 19]. p27Kip1 is a regulator of cyclin-dependent kinase activity and an inhibitor of G1-S cell cycle progression. Fang et al. [20], in a study with mice presenting with autoimmune disease, found that cell proliferation occurs by downregulating antiproliferative molecules, p21 and p27. The need for clarification regarding the development of autoimmune thyroid disease in hyperthyroidism justifies studies on the mechanisms of cell protection and death of thyrocytes and intrathyroidal lymphocytes in GD, as well as the action of thionamides. This study is aimed at evaluating antiapoptotic, proapoptotic, and cell proliferation markers in GD by using histopathological samples of thyroidectomy products in a representative number of patients associated with clinical and laboratory variables, as well as medications.

## 2. Materials and Methods

### 2.1. Study Design

The study was carried out at the University of Campinas Teaching Hospital. Paraffin blocks of thyroidectomy products with anatomopathological and clinical diagnoses of GD and normal thyroid were selected. New sections were prepared for the immunohistochemical analysis of markers of apoptosis (Fas, Fas ligand, and BID), antiapoptosis (BCL-2, MCL-1), cell proliferation (Ki-67), and antiproliferation (p27Kip1) in thyroid cells and intrathyroidal lymphocytic infiltrate, as well as an evaluation of the lymphocytic infiltrate pattern in haematoxylin-eosin (HE) stained cells by an experienced pathologist. Clinical and laboratory parameters such as age, sex, serum thyroid-stimulating hormone (TSH) and free levothyroxine (FT4) concentrations, and antithyroperoxidase (anti-TPO) and antithyroglobulin (anti-Tg) antibodies were collected, retrospectively, by reviewing the chart.

The institutional review board approved the study under the opinion number 783.083, and no competing financial interests exist.

### 2.2. Patients

We selected 67 individuals from an iodine area who underwent thyroidectomy between 1996 and 2015 and whose specimens were maintained by the tissue bank of the hospital; 53 were GD patients, and 14 had normal thyroid samples in the control group. Out of 67 individuals studied, 52 were female. The clinical diagnosis of GD was confirmed based on the presence of goiter, hyperthyroidism, or euthyroidism using thionamide, with at least one of the antithyroid serum antibodies in high concentrations. The indications of thyroidectomy were difficult disease control even in optimized therapy or serious adverse reactions due to the use of antithyroid drugs such as hepatitis drug, cutaneous reaction, or agranulocytosis. We included in the GD sample, eight patients who were not using ATD at the time of surgery. Among patients with GD, 54.72% were smokers, 88.68% were using thionamide at the time of surgery, 84.91% had exophthalmos, and only 11.33% underwent previously radioiodine therapy. In the control group, the indications of thyroidectomy were thyroid infiltrative malignant neoplasm of larynx or suspicion of thyroid neoplasia. The inclusion criteria were the absence of antithyroid antibodies, euthyroidism, and normal thyroid tissue by anatomopathological analysis. If there were thyroid neoplasia in the sample, that case was excluded.

### 2.3. Laboratory Evaluation

Serum TSH concentrations were measured by the electrochemiluminescence (EIA) method—a sandwich technique used in the Elecsys TSH immunoassay analyser (Roche), and a range of 0.4 to 4.5 *μ*IU/mL was considered normal. The concentrations of FT4 were measured by the EIA method—the competition principle used in the Elecsys FT4 immunoassay analyser (Roche), with a normal range of 0.9 to 1.8 ng/mL. Anti-TPO and anti-Tg antibodies were measured by EIA, the competition principle used in the Elecsys anti-TPO and anti-Tg (Roche) immunoassay analyser, with normal values lower than 35 *μ*IU/mL and 115 *μ*IU/mL, respectively.

### 2.4. Tissue Specimens

We selected 10% formalin-fixed paraffin-embedded blocks of 67 thyroidectomies. We performed new histological sections for lymphocytic infiltrate evaluation and immunohistochemical analysis with proliferation, antiproliferation, cell apoptosis, and antiapoptotic markers.

### 2.5. Immunohistochemistry

The selected paraffin blocks were submitted to 4 *μ*m slices in a Leica® RM2125RT microtome. Tissue sections were deparaffinized in xylene and dehydrated in graded ethanol series, washed with distilled water, and then placed in a pressure cooker in Tris-EDTA buffer pH 8.9 for 5 minutes at 95°C (CD 95, Fas ligand, Ki-67, and BCL-2) or citrate buffer pH 6.0 (BID, MCL-1, and p27). Endogenous peroxidase and nonspecific binding were blocked with 0.3% hydrogen peroxide in methanol for 15 minutes. After rinsing in phosphate-buffered saline at pH 7.2, 10% bovine serum (Wako, Osaka, Japan) was applied for 20 minutes. Sections were then incubated with primary antibodies overnight at 4°C. The primary antibodies and positive controls within each marker used are shown in Table 1.

Anti-MCL-1 monoclonal antibody detects the long chain of MCL-1 protein (37 kDa), called isoform 1, and is defined as an antiapoptotic protein [21]. A second-generation peroxidase-based detection system (Advance, Dako) was used to visualize antigen-antibody reactions.

### 2.6. Immunohistochemical Evaluation

All analyses were performed under light microscopy (Eclipse E200, Nikon Instruments Inc., NY, USA). Two blinded observers, one pathology expert in thyroid (IBS) and the first author (JCV), reviewed all thyroid slides at different times individually (Cohen's kappa = 0.628 − substantial agreement), and cases with inconsistent scores were reevaluated to achieve a consensus score. An evaluation of semiquantitative and visual immunohistochemistry (IHC) was done in follicular epithelium and lymphoid follicles, in each case, in at least three representative tissue areas, and different cut-off points were used for each marker, based on the extent of expression at 40x magnification (calculating the percentage of immunoreactive cells of at least 500 cells). For cell proliferation evaluation, we used Ki-67 and p27kip1, with nuclear positivity on thyrocytes and lymphocytes. For apoptosis, we used BID, Fas, and FasL with cytoplasm positivity in thyroid cells and cytoplasm or nuclear membrane positivity in lymphocytes. For antiapoptosis, we used BCL-2 and MCL-1, with cytoplasm positivity in thyroid cells and lymphocytes. It was considered strong expression of Fas/FasL when a minimum of 50% of cells were clearly positive. The staining intensity of Ki-67 was scored as expression less than 1% and greater than 1%. The marker BID was evaluated and classified as expression lower and greater than 50%. For BCL-2, the staining was divided into no or weak reactivity (0–25%) and positive (>25%). For p27Kip1, the expression was classified into three groups: no expression (0%), weak positivity (1 to 25%), and strong (>25%) positivity. MCL-1 protein expression was classified as strong positivity (>20%) and weak positivity (<20%).

### 2.7. Statistical Analyses

Aiming to describe the sample profile according to the variables studied, we used frequency tables of categorical variables (sex, presence of lymphocytic infiltrate, and antithyroid antibodies in the two groups, as well as exophthalmos and radioiodine treatment only in cases of GD) with absolute frequency (*n*) and percentage (%) values, as well as descriptive statistics of numerical variables (TSH, free T4, goiter volume, and age), with mean values, standard deviation, minimum, maximum, and median values. For comparing categorical variables, the chi-square test was used and, when necessary, Fisher's exact test was used as well. For numerical variables, the Kruskal-Wallis test was used, followed by Dunn's post hoc test and the Mann–Whitney test. The level of significance adopted for the study was 5%. For statistical analysis, the following computer program was used: SAS System for Windows (Statistical Analysis System), version 9.4. SAS Institute Inc., 2002–2008, Cary, NC, USA.

## 3. Results

The younger patients were those with GD (mean 39.51 years, *p* = 0.0021) who also had the lowest median TSH levels (0.01, *p* = <0.0001) and a higher goiter volume (59.6 mL, *p* = 0.0128). Most patients (77.36%) underwent total thyroidectomy due to the difficulty of controlling hyperthyroidism.

### 3.1. Relationship between Lymphocytic Infiltrate and the Presence of Antibodies in Graves' Disease

**Figure 1:**
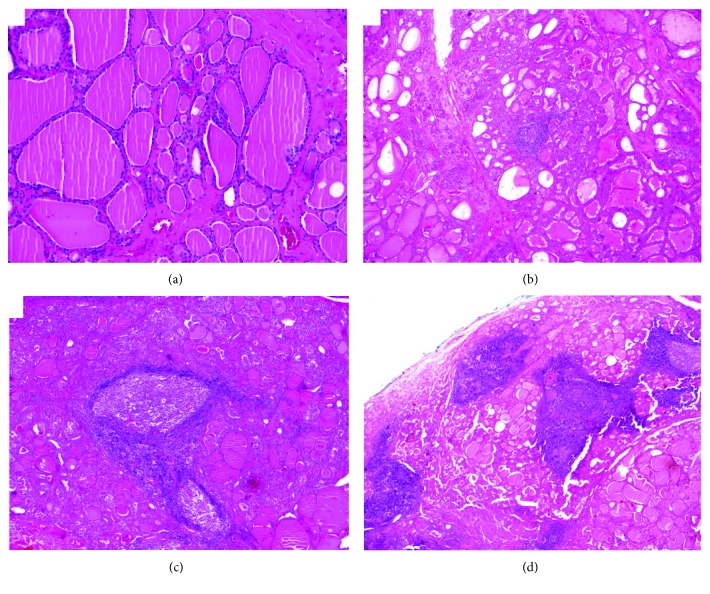
Lymphocytic infiltrate degrees: (a) without infiltrate or infiltrate 0 (×10); (b) light grade or focal infiltrate (×10); (c) moderate degree infiltrate or 2 (×10); (d) intense degree infiltrate or 3 (no magnification).

**Figure 2:**
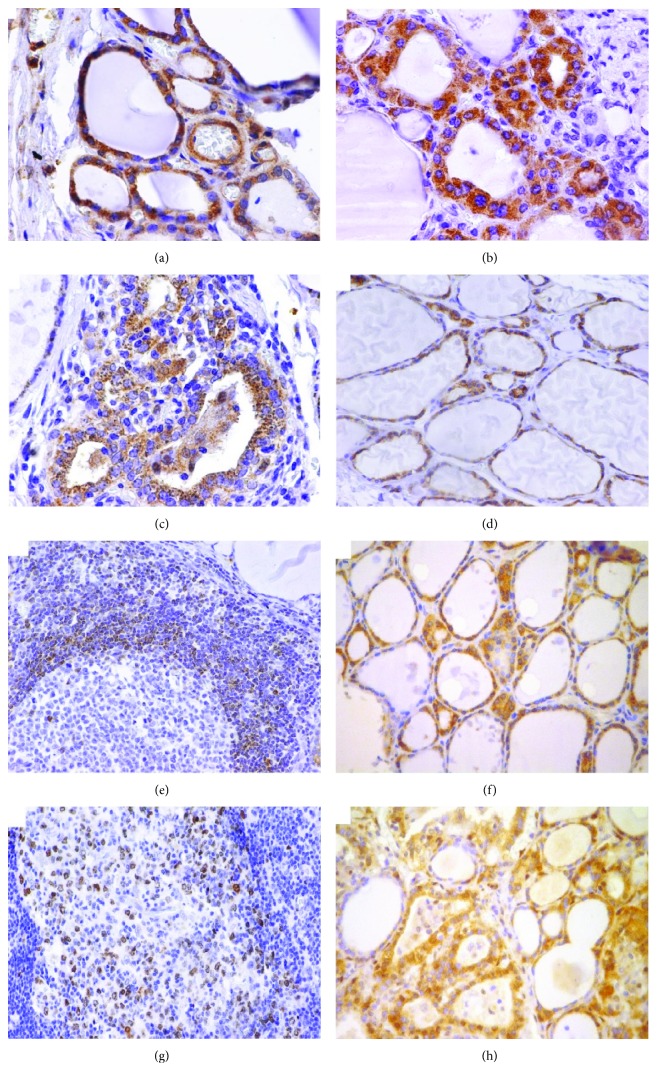
Expression of markers in thyroid specimens. (a) Fas (×40); (b) FasL (×40); (c) BID (×40); (d) BCL2 (×10); (e) BCL2 in intrathyroidal secondary lymphoid follicle (×40); (f) MCL1 (×10); (g) Ki67 in intrathyroidal secondary lymphoid follicle (×40); (h) p27Kip1 (×10).

We could observe that as lymphocytic infiltrate (Figure 1) in GD becomes more pronounced, the frequency of antibody positivity (Figure 2) increases and, in the case of antithyroglobulin antibody, it was significant: those with negative lymphocytic infiltrate did not have antibody positivity and among those with severe lymphocytic infiltrate, all had antibody positivity (*p* = 0.0003).

### 3.2. Immunohistochemical Analyses

#### 3.2.1. Proliferation and Antiproliferation Cell Markers

*(1) Analysis of Graves' Disease Patients and the Control Group*. The immunostaining results of proliferative cell markers in GD patients and the control group are summarized in Table 2 and Figure 1.

*(2) Analysis of Graves' Disease Patients*. We could observe that six patients with Graves' disease who submitted to radioiodine therapy (RIT) presented lower lymphocytic expression of the p27 marker (<25%) and those who took beta-blockers, such as propranolol, had lower Ki-67 expression in thyrocytes than those who did not (Table 3).

#### 3.2.2. Proapoptotic Markers

*(1) Analysis of Graves' Disease Patients and the Control Group*. Patients with GD using ATD have a higher expression of the proapoptotic marker BID in thyrocytes (expression greater than 50% in 72% of cases of each disease), whereas in the control group, the expression was less than 50% in most individuals, 64% (*p* = 0.0318).

*(2) Analysis of Graves' Disease Patients*. Patients with GD using beta-blocker drugs have higher expression of the proapoptotic marker BID in thyroid cells (*p* = 0.0432), as shown in Table 4.

If we divided these patients according to the use of ATD, those who were not using ATD had higher expression of the proapoptotic marker BID (greater than 50%) in intrathyroid lymphocytes (*p* = 0.0094), as shown in Table 4.

#### 3.2.3. Antiapoptotic Markers

*(1) Analysis of Graves' Disease Patients and Control Group*. The antiapoptotic marker MCL-1 is expressed more in thyrocytes (expression greater than 20%) in cases of GD using ATD (95.45%) than that in control subjects (64.29%), with a *p* value of 0.0117.

*(2) Analysis of Graves' Disease Patients*. The expression levels of antiapoptotic markers BCL-2 and MCL-1 in intrathyroid lymphocytes are lower in patients who took beta-blocking drugs at the time of thyroidectomy, as shown in Tables 5 and 6. It was also observed that the lowest expression of MCL-1 protein (<20%) in intrathyroid lymphocytes occurred in patients with the largest thyroid volume (mean 97.92 cm^3^), despite having a large standard deviation (±82.61 cm^3^) with *p* = 0.0229.

## 4. Discussion

GD pathophysiology involves dysregulation of apoptosis and lymphocytic infiltration, in addition to the result of the balance between proapoptotic and antiapoptotic factors, as well as proliferative and antiproliferative factors in thyroid cells and intrathyroidal lymphocytes [22].

When analysing the expression of markers between GD patients and the control group, we found that Ki-67 (proliferation) and p27Kip1 (antiproliferation) expression in intrathyroidal lymphocytes was lower in the control group than that in GD patients using antithyroid drugs (ATD). Studies have shown a tendency toward apoptosis of intrathyroidal lymphocytes in patients with GD who take ATD and exhibit less cell proliferation, with an elevation of the p27Kip1 marker and its regulator Ki-67 [23]. BID expression in thyrocytes was greater in patients with GD using antithyroid drugs than that in the control group, showing the proapoptotic effect of these drugs. The MCL-1 marker exhibited higher expression in thyrocytes of patients with GD using ATD than that in the control group, indicating that there is strength in the body to prevent apoptosis of these cells, promoted by the marker of apoptosis BID, as cited above.

According to the literature [24], we found a positive association between patients who received radioiodine treatment and the lowest expression of marker p27 in intrathyroidal lymphocytes; that is, lymphocytes cause a greater inflammatory reaction, increased apoptosis in thyrocytes, and a reduction of goiter volume. Patients with GD use medications such as thionamide, beta-blockers, and iodine solution to control thyrotoxicosis and prepare for surgery. In our study, the use of beta-blockers was associated with the greater expression of BID (proapoptotic) and lesser expression of Ki-67 (cell proliferation) in thyrocytes [25], suggesting that these medications may stimulate apoptosis of thyrocytes, reducing the cell proliferation rate and helping in disease control.

We divided GD patients into two groups: with and without the use of ATD at the time of surgery. The expression of BID in intrathyroidal lymphocytes was lower in patients using ATD, suggesting that they had less lymphocytic apoptosis, which stimulates less activity of thyrocytes and may be another disease control mechanism of ATD.

In relation to MCL-1 in GD, patients with the lowest expression of this marker in lymphocytes are those with the largest goiters; those using beta-blockers also had lower expression of MCL-1 and BCL-2 in lymphocytes. If MCL-1 or BCL-2 is decreased in lymphocytes, they survive less and have less stimulating thyrocyte apoptosis, leading to a greater goiter volume. There are already studies evaluating melanoma and thyroid carcinoma [25, 26] showing the reduction of BCL-2 and MCL-1 expression in people using propranolol.

The higher proportion of positive detectable antithyroid antibodies found in patients with GD with more prominent lymphocytic infiltrate suggests that a larger lymphocytic infiltrate and activation of T cells led to a higher recruitment of B cells and the development of immune response via antithyroid antibodies [27].

The study limitations were as follows: the impossibility to show a predominance of one marker over another. We evaluated the expression of markers separately, and there was a lower number of individuals in the control group due to the lower indication of thyroidectomy in these cases. The strengths were as follows: the study was carried out with anatomopathological findings, thyrocytes, and intrathyroidal lymphocytes; the study contained many patients and immunohistochemical markers, as well as an evaluation of drugs in use and their effects on apoptotic mechanisms and cell proliferation.

In conclusion, the recognition of thionamides as immunomodulatory drugs, with alteration of the lymphocytic infiltrate in patients who use them, and RIT as a lymphocyte sparer represents significant findings obtained from this study. We believe that these findings in the immunohistochemical expression of cell antiproliferation (p27Kip1 on intrathyroidal lymphocytes), apoptotic (BID on thyroid cells and intrathyroidal lymphocytes), and antiapoptotic (MCL-1 on thyroid cells and intrathyroidal lymphocytes) markers may be relevant in the development of new drugs and a better understanding of the pathophysiology of these diseases. Additionally, understanding the role of beta-blockers, more specifically propranolol, in GD has been poorly explored in thyroid studies, although it has been described as an important aid in melanoma cells and cardiac cells. Therefore, the greater BID (apoptotic) expression in the thyrocytes and lower expression of MCL-1 (antiapoptotic) in lymphocytes may lead to the routine use of these drugs in therapy for Graves' disease, changing its indication. Further studies are needed to better understand the pathophysiology of these diseases and to better target the therapeutic regimen for less need for surgeries and complications.

## Figures and Tables

**Table 1:** Characteristic of primary antibodies.

Marker	Antibody	Dilution	Positive control	Producer
BCL-2	Mouse monoclonal, clone 124	1 : 150	Colon carcinoma	Dako, USA
MCL-1	Rabbit monoclonal, clone Y37	1 : 50	Tonsil	Abcam, Cambridge, USA
FasL	Rabbit polyclonal	1 : 70	Tonsil	Abcam, Cambridge, USA
Fas	Rabbit polyclonal	1 : 25	Tonsil	Abcam, Cambridge, USA
BID	Rabbit monoclonal, clone Y8	1 : 150	Prostate carcinoma	Abcam, Cambridge, USA
Ki-67	Mouse monoclonal, clone MIB-1	1 : 500	Lymph nodes	Dako, CA, USA
p27Kip1	Mouse monoclonal, clone SX53G8	1 : 50	Tonsil	Dako, USA

**Table 2:** Expression of proliferation (Ki-67) and antiproliferation (p27) cell markers in thyrocytes and lymphocytes according to the diagnosis: Graves' disease versus normal control (chi-square test/^∗^Fisher's exact test).

Markers	%	Graves	Control	*p* value
Ki-67 in thyrocytes	<1%	56.82%	85.71%	0.1254
	>1%	43.18%	14.29%	
Ki-67 in lymphocytes	<1%	38.64%	71.43%	0.0001
	>1%	61.36%	28.57%	
p27 in thyrocytes	<25%	18.18%	21.43%	0.6892^∗^
	>25%	81.82%	78.57%	
p27 in lymphocytes	<25%	63.64	92.86%	<0.0001^∗^
	>25%	36.36%	7.14%	

**Table 3:** Expression of proliferation markers in thyrocytes and lymphocytes in patients with Graves' disease according to the type of treatment (chi-square test/^∗^Fisher's exact test).

Markers	Cells		ATD (%)	Without ATD (%)	RIT (%)	Beta-blocker (%)
Ki-67	Thyrocytes	<1%	56.82	25	83.33	**62.16**
		>1%	43.18	75	16.67	**37.84**
		*p* value	0.1337		0.1919^∗^	**0.0385**
	Lymphocytes	<1%	38.64	37.5	66.67	45.95
		>1%	61.36	62.5	33.33	54.05
		*p* value	1.0		0.1710^∗^	0.0608

p27	Thyrocytes	<25%	18.18	50	50	29.73
		>25%	81.82	50	50	70.27
		*p* value	0.0715		0.1480^∗^	0.1433^∗^
	Lymphocytes	<25%	63.64	37.5	**100**	64.86
		>25%	36.36	62.5	**0**	35.14
		*p* value	0.4115		**0.0445** ^∗^	0.4798

**Table 4:** Expression of apoptotic markers in thyrocytes and lymphocytes in patients with Graves' disease according to the type of treatment (chi-square test/^∗^Fisher's exact test).

Markers	Cells		ATD (%)	Without ATD (%)	RIT (%)	Beta-blocker (%)
BID	Thyrocytes	<50%	27.27	37.5	16.67	**18.92**
		>50%	72.73	72.5	83.33	**81.08**
		*p* value	0.6756		0.6627^∗^	**0.0432** ^∗^
	Lymphocytes	<50%	**97.73**	**62.5**	100	97.30
		>50%	**2.27**	**37.5**	0	2.70
		*p* value	**0.0094**		1.0^∗^	0.0770^∗^

Fas	Thyrocytes	<50%	2.27	0	0	2.70
		>50%	97.73	100	100	97.30
		*p* value	—		—	—
	Lymphocytes	<50%	65.91	32.5	83.33	**70.27**
		>50%	34.09	62.5	16.67	**29.73**
		*p* value	0.2346		0.3794^∗^	**0.0421**

Fas-L	Thyrocytes	<50%	2.27	12.5	0	0
		>50%	97.73	87.5	100	100
		*p* value	0.2866		—	—
	Lymphocytes	<50%	72.73	75	100	72.97
		>50%	27.27	25	0	27.03
		*p* value	1.0		0.1639^∗^	1.0^∗^

**Table 5:** Expression of antiapoptotic markers in thyrocytes and lymphocytes in patients with GD according to the type of treatment (chi-square test/^∗^Fisher's exact test).

Markers	Cells		ATD (%)	Without ATD (%)	RIT (%)	Beta-blocker (%)
BCL-2	Thyrocytes	<30%	6.82	25	16.67	13.51
		>30%	93.18	75	83.33	86.49
		*p* value	0.1643		0.4952^∗^	0.3049^∗^
	Lymphocytes	<30%	84.09	87.5	100	**91.89**
		>30%	15.91	12.5	0	**8.11**
		*p* value	1.0		0.5742^∗^	**0.0358** ^∗^

MCL-1	Thyrocytes	<20%	4.55	25	16.67	8.11
		>20%	95.45	75	83.33	91.89
		*p* value	0.1072		0.4175^∗^	1.0^∗^
	Lymphocytes	<20%	75	37.5	100	**81.08**
		>20%	25	62.5	0	**18.92**
		*p* value	0.0889		0.1588^∗^	**0.0069** ^∗^

**Table 6:** Comparison of markers of cell proliferation and apoptosis among patients with Graves' disease according to radioiodine, thionamide, and beta-blocker therapy.

Treatment		Ki-67	p27	BCL-2	MCL-1	BID	Fas
RIT	Thyrocytes						
	Lymphocytes		↓				
ATD use	Thyrocytes						
	Lymphocytes					↓	
Beta-blocker use	Thyrocytes	↓				↑	
	Lymphocytes			↓	↓		↓
